# Scientific News

**Published:** 1879-12

**Authors:** 


### SCIENTIFIC NEWS.

The following editorial from the *Popular Science Monthly* will be of interest to many readers of the REGISTER, who undoubtedly felt an interest in the late Professor Vaughan, at one time Professor of Chemistry in the Ohio Dental College:

"We have received various communications from widely different and distant sources in relation to the reputation and works of the late Professor Daniel Vaughan. Severe animadversions have been passed upon the depreciatory tone of comment that has been indulged in with regard to his personality and life; and there has been inquiry as to where his writings may be obtained. Several suggestions have been made respecting the publication of an edition of the most important and popular of his scientific contributions. A correspondent of Salem, Massachusetts, suggests that a very attractive and valuable volume could be made up of his papers on "The Tides," "The Rings of Saturn," "The Origin and End of the World," "The Advent and Appearance of New Stars," "The Nebular Hypothesis," "The Plurality of Worlds," "The Primitive Earth," "The Ancient Atmosphere," "Physics of the Internal Earth," "Volcanoes," "The Moon," "Revelations of Spectrum Analysis," and the "Catastrophes in Celestial Space." These are certainly interesting topics, and they were handled by Professor Vaughan, not only with the ability of an able expositor, but with the freshness of an independent thinker, who had formed his own opinions upon many of the questions involved.

"Professor Vaughan, as we, however, understand, left no property to pay for the publication of his works, and whether such a volume can be issued will depend upon how publishers regard the venture, or whether he has any friends sufficiently interested in his memory and productions to co-operate in bringing out a collection of his essays."

In 1859 Prof. Vaughan published a small collection of papers entitled "Popular Physical Astronomy," containing some of those already mentioned, but at this time it is out of print. The suggestion of the Salem correspondent is a good one, and should meet with material approval from all who take pride in American scientific progress.

---

THE first paper of the last day of the meeting of the National Academy was bv Prof. Joseph LeConte, on the glycogenic function of the liver. It was read by Dr. George T. Barker, and was a continuation of the paper read at the previous meeting of the Academy. Dr. LeConte contended that the chief function of the liver is in preparing sugar to be oxydized in the capillaries, whither it is carried by the blood. He regarded the liver also as a sort of storehouse for fuel; the carbon received one day may be held until the next day, when it is oxydized in the capillaries in contact with the tissues, with the evolution of heat.—*Scientific American.*

---

DR. C. HUTER, of Greifswald, has invented a process he calls "cheiloangioscopy" by which the flow of blood in the vessels of the human subject may be closely observed. The person's head is placed in a frame something like that used by photographers, and to which is attached a lamp and microscope; the lower lip drawn out and held to the microscope stage, and while a condenser floods it with light, the lip with its blood vessels can be examined with a low power objective. The value of this process will be inestimable to the physician in forming his diagnosis of disease.

---

BERTHELOT has succeeded in making alcohol by immersing in an aqueous solution of glucose, a pair of sponge-platinum cylinders, through which are passed in rapid succession alternate currents of positive and negative electricity.

---

MR. HEPBURN, in a paper read before the Odontological Society of London, says "that the direct action of nicotine on the teeth is decidedly beneficial. The alkalinity of the smoke must necessarily neutralize any acid secretion which may be present in the oral cavity, and the antiseptic property of the nicotine tends to arrest putrefactive changes in carious cavities."

---

The use of carrier-pigeons in the British meteorological service has been suggested, for bringing accounts of the weather from points three hundred to five hundred miles in the Atlantic Ocean. This would in a measure give notice of storms that otherwise arrive unannounced upon the European coast.

---

The work upon the artesian well near Buda Pesth, Hungary, commenced in 1868, is now completed. It is the deepest well in the world, reaching a depth of thirty-two hundred feet, the temperature of the water from it being one-hundred and sixty-five degrees Fahrenheit.

---

With compound sulphide of carbon prisms, M. Thollon produced a map of the solar spectrum ten metres long, and containing nearly four thousand lines.

---

Bernhard von Cotta, of Saxony, an eminent geologist and professor of geology in the University of Freiberg, died at that place September 14th, aged seventy-one years.

---

Prof. B. F. Mudge places the existence of man on the earth at two hundred thousand years.

---

The production of honey in this country is estimated at thirty-five million pounds per annum.

Among the latest German patent applications there is one for the process of making a green color by oxidizing the sulphide of tetramethyldiamidotriphenylmethan.—*Scientific American.*

